# Stress-related asthma and family therapy: Case study

**DOI:** 10.1186/1744-859X-11-28

**Published:** 2012-11-13

**Authors:** Maria Theodoratou-Bekou, Ourania Andreopoulou, Panoraia Andriopoulou, Beatrice Wood

**Affiliations:** 1 T.E.I. of Patras, School of Health and Welfare Professions, 20, Stavropoulou str, Patras, 25002, Greece; 2 Medical School, University of Patras, Rio, Greece; 3 New York College, 21, Votsi str, Patras, 22621, Athens, Greece; 4 Women & Children’s Hospital of Buffalo, University of Buffalo, 219 Bryant Street, Buffalo, NY, 14222, USA

**Keywords:** Family, Differentiation, Asthma, Intervention

## Abstract

This paper applies the Biobehavioral Family Model (BBFM) of stress- related illness to the study and treatment of an adolescent with intractable asthma. The model is described, along with supportive research findings. Then a case study is presented, demonstrating how the model is clinically applied. We tell the story of an asthmatic adolescent presenting for therapy due to her intense asthmatic crises, and the case is presented to exemplify how the BBFM can help understand the family-psychobiological contribution to exacerbation of disease activity, and therefore guide treatment towards the amelioration of severe physical symptoms. Facets of the patient’s intra-familial interactions are consistent with the BBFM, which support clinical validation of the model. In the case described, it is likely that additional asthma medications would not have had the desired ameliorative effect, because they did not target the family relational processes contributing to the symptoms. The recognition of the influences of family relational processes on the disease was crucial for effective intervention. The therapy incorporates and weaves together BBFM understanding of family patterns of interaction and physiological/medical concerns integrated with Bowenian intervention strategies. This case study validates the importance and usefulness of BBFM for intervention with stress-sensitive illnesses such as asthma.

## 

Research has demonstrated that the family plays a decisive role in physical as well as emotional health [1]. Families and social systems constitute complex, dynamic ecologies that are essential contexts for mind–body inter-relations. Understanding health and illness therefore requires an investigatory paradigm that incorporates these ecologies.

Minuchin and colleagues [2] in their psychosomatic family model addressed the issue of the contribution of socio-relational factors in “psychosomatic” health problems, by identifying specific family relational processes that were associated with, and were proposed to affect, as well as be affected by, disease activity. According to their model, the development and maintenance of psychosomatic symptoms in children are attributed to specific family interaction patterns in the context of physiological vulnerability of the child. The model spawned many advances in family-based theory and practice in the medical arena. It is currently seen to be most relevant for children and adolescents with somatizing disorder. The Biobehavioral Family Model (BBFM) [3] had its origins in the Psychosomatic Family model. However, there are crucial distinctions between the two models. The Psychosomatic Family model presented a type of family. That is, the Psychosomatic Family was characterized by enmeshment, rigidity, overprotection, with poor conflict resolution and triangulation of the ill child in conflict. A given family was either a “psychosomatic family” or not a “psychosomatic family”. In contrast, the BBFM is a model of the configuration of several family dimensions which are proposed to interact in more complex and varied ways, and with more nuanced effects on child emotional and physical wellbeing. There is no such thing as a “biobehavioral family”. Rather, the BBFM is a model of the interaction and effect of various configurations of family relational processes.

The BBFM was originally developed to understand how family process influences chronic illnesses, such as inflammatory bowel disease [4] and asthma [5], however it is now more broadly applied to ‘stress-related illness’ [6]*. (The term stress-related illness could be defined as follows: The appearance of clinical symptoms of disease, as the organism’s response to stressful conditions or stressors, consisting of a pattern of physiological and psychological reactions, both immediate and delayed*[7]*).*

The BBFM is a biopsychosocial model, which posits that particular family relational patterns influence and are influenced by the psychological and physiological processes of individual family members. The model proposes multiple pathways through which family relational stress and emotions may influence a child’s health [8].

## A Psychosocial-Mind–body continuum of disease

The BBFM model rests upon a systems conceptualization of disease, one that embraces the psychosocial-mind–body perspective. The conceptualization features a continuum of psychological and physical disease, which varies according to the relative proportions of psychosocial and biological influence on the disease process.

Both physical and psychological disease can be conceptualized in terms of a continuum of relative contribution of psychological and biological (i.e., mind–body) influence. *Psychosocial and biological factors interact as they influence a disease.* They represent mechanisms or pathways of biobehavioral influence. The pathways are assumed to be bidirectional, such that having a disease elicits psychosocial and emotional stress, and psychosocial and emotional stress evokes the disease process. BBFM specifies pathways by which the family plays a pivotal role in these psychobiological processes.

Consistent with the biobehavioral continuum of disease presented above, the BBFM is broadly applicable to any disorder, psychological or physical, in which there is a psychobiological pathway.

## The Biobehavioral family model of stress related illness

**Figure 1:**
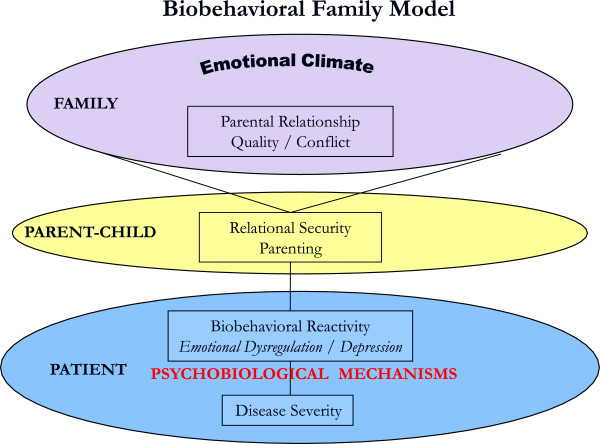
Biobehavioral Model (Wood et al.,2000, 2008).

The BBFM posits that family emotional climate, quality of parent-parent relations, parent–child relational security and biobehavioral reactivity (emotion regulation/ dysregulation) are processes that influence one another and collectively either buffer or potentiate psychobiological processes influencing disease activity in stress-related illnesses [8]. The model assumes that directions of causal effect are circular, but the current focus is on the child’s physical and emotional outcomes (see Figure 1).

Family emotional climate refers to the overall intensity and valence of emotional exchange. It colours all aspects of family relationship, and therefore it is probably a key factor contributing to emotional status and outcomes in family members. A negative family emotional climate includes hostility, criticism, verbal attacks, etc., and is similar to the criticism construct of Expressed Emotion (EE). Positive aspects include warmth, affection, support, affirmation, etc. Family emotional climate is characterized by the intensity and balance of negative and positive emotional exchange among family members. This balance or imbalance can be construed as reflecting one aspect of family-level emotion regulation or dysregulation.

*Several family relational process factors contribute to family emotional climate. For example, proximity, which is defined as the extent* to which family members share personal space, private information, and emotions. Generational hierarchy refers to the extent to which caregivers are in charge of the children by providing nurturance and limits through strong parental alliance and absence of cross-generational coalitions. Proximity and Generational Hierarchy derive from Wood’s theoretical differentiation of the Minuchin’s construct of “boundaries” [4]. Parental relationship quality refers to interaction patterns which include mutual support, understanding and adaptive disagreement (respectful and resolving) versus hostility, rejection and conflict. Parental discord not only contributes to negative family emotional climate, but also has direct effects on children’s emotional functioning, with emotion dysregulation (e.g. anxiety, depression, etc.) mediating the link between parental conflict and disease activity. Triangulation [9] refers to the involvement of a child in the parental conflictual process in ways that render the child responsible, blamed, scapegoated, or in loyalty conflict. Responsivity refers to the extent to which family members are behaviorally, emotionally, and physiologically responsive to one another. Responsivity depends, in part, on the biobehavioral (i.e., emotional) reactivity of each family member. Moderate levels of emotional/physiological responsivity allow for empathic response among family members. Extremely high levels of responsivity can exacerbate maladaptive emotional/physiological resonance in the family; possibly worsening psychologically influenced emotional or physical disorders. Extremely low levels of responsivity may be part of a general pattern of neglect or avoidance, leaving family members vulnerable to internal, familial, or environmental stressors [5]. Responsivity is similar to the dimension of enmeshment and disengagement [3], but it includes biologically predisposed aspects of temperament, as well as environmentally derived aspects of emotion regulation.

Relational Security is a construct related to attachment. The importance of integrating attachment and family systems theory is evidenced by recent theory and research. (Also see Wood, 2002 [10] for a multi-author special issue devoted to the integration of attachment and family systems theory and research). Attachment security is also a key factor in emotion regulation. The BBFM proposes that parent–child relational security mediates and/or moderates the impact of stressful family process (or life events) on disease-related emotional a physiological regulation processes in children. Empirical findings support this hypothesis [1,8].

Biobehavioral reactivity is a pivotal construct in the BBFM, linking psychological and emotional processes to disease processes. It is conceptualized as the intensity and manner in which an individual family member responds physiologically, emotionally, and behaviorally to emotional stimuli. Behavioral reactivity comprises both emotional and physiological aspects of emotion regulation or dysregulation. Emotion regulation is accompanied by relatively stable physiological regulation, whereas emotion dysregulation is accompanied by physiological dysregulation. Thus, emotion regulation buffers, while emotion dysregulation transmits (or escalates) the effect of stress and emotional challenge to disease processes through psychobiological pathways. Biobehavioral reactivity involves hypothalamic-pituitary-adrenal axis (HPA), autonomic nervous system processes, neuroendocrine and immune processes. Depending on which physiological processes are activated or deactivated during patterns of emotion dysregulation, such processes may influence physical and emotional disorders depending upon the disease-specific psychobiological pathway by which such effect can take place [1]. Biobehavioral reactivity thus transmits the effect of family relational patterns upon the child.

## Empirical validation of the BBFM

To date no single study has tested the BBFM in its entirety, but studies of children with inflammatory bowel disease, epilepsy, Psychogenic Non-epileptic Seizure Attacks, PNEA and asthma [1,5,11] report findings consistent with several links posited in the model, thus supporting it as a heuristic model. The rest of this paper will illustrate the utility of the BBFM as a clinical model in the treatment of an adolescent with asthma, integrated with Bowenian based intervention.

## The fit of BBFM and Bowenian constructs

It is noteworthy that BBFM conceptualizations are consistent with ‘Family Systems Theory’ introduced by Murray Bowen [9]. Bowen’s theory features ‘processes going on in the relationship space between a person, that is the interpersonal emotional processes. The BBFM does as well, however it also explicitly identifies specific relational aspects of emotional process. Furthermore as a configurational conceptualization, the BBFM describes not only the specific components that constitute the family emotional climate, but also how they interact with one another. Bowen’s concepts are highly compatible with the BBFM and, in addition, also bring to bear a crucial developmental perspective.

"Furthermore, we would like to clarify the uniqueness of the notion ‘Family systems theory’. In this article, the term ‘Family systems theory’ refers to the Bowen family systems theory. Bowen theory is essentially different from most other family approaches as Kerr (1981), Bregman 2004), among others, argue. It is a theory about human behavior that is rooted in thinking about natural systems. Bowen family systems theory is about the emotional functioning of the human species. The theory postulated that the human family is a multigenerational, natural, living system and that the emotional functioning of each member of the system affects the functioning of the other members in predictable ways (Bowen [12] , 1978, Comella [13] , 2000)."

"The term ‘psychosomatic family model’ refers to a different theoretical model. Minuchin, Rosman, and Baker’s open systems model of the “psychosomatic family” comprises a constellation of family patterns of functioning and somatic patterns of disease activity and illness behavior in chronically ill children (1975)."

Bowen introduced the notions of emotional forces for ‘togetherness’ and differentiation’. The togetherness amalgam is bound together by assigning positive value to thinking about the other before self, living for the other, sacrifice for other, love and devotion and compassion for others, and feeling responsible for the comfort and wellbeing of others’. In contrast, “the differentiating force places emphasis on “I” in defining the foregoing characteristics. It is the ‘responsible I’ which assumes responsibility for one’s own happiness and comfort and avoids making demands on others”. Differentiated self is defined as: “one who can maintain emotional objectivity, while at the centre of an emotional system in turmoil and, at the same time, actively relating to the important persons of the system”.

Problems occur when differentiation is compromised by various family and individual factors. Bowen identified a number of mechanisms by which families transmit problematic emotional processes across generations: the family projection process, emotional cut-off, and levels of parental differentiation. Bowen’s conceptualizations also are important because they offer internal mechanisms by which family interactions and emotions may influence the individual’s body state. Bowen suggested that many of the family emotional processes reflect individuals’ unconscious, anxiety-driven survival responses, responses that involve emotional and bodily processes together. Bowen calls these automatic responses ‘emotional reactivity’. Individuals whose behavior is characterized by high emotional reactivity are considered to be poorly ‘differentiated’: they find it difficult to think explicitly about their behavior and feelings (feelings are the conscious awareness of emotional states) and also to distinguish their thoughts and feelings from those of others within a relationship system. These notions tie in well with ‘Responsivity’, a key construct in Wood’s model. Responsivity is conceptualized at both family and individual levels. At the family level, interpersonal responsivity is defined as the degree or intensity with which people respond physiologically, emotionally, and behaviorally to one another. At the individual level, responsivity influences, and is influenced by, biobehavioral reactivity, defined as the degree of intensity with which an individual responds physiologically to stimuli [9].

While Bowen’s model specifies internal processes influencing individual physiology, Wood’s model [11] identifies specific pathways by which family relational process may influence the physiology of the individual’. The models fit together well, as they illustrate complementary pathways and mechanisms by which family relational patterns and psychosomatic symptoms co-occur. Therefore, by using the BBFM to clarify the specific family relational factors that contribute to the illness, Bowenian interventions addressing differentiation can be more precisely targeted to family-specific relational factors.

### Pediatric asthma

Asthma is one of the most common diseases in childhood, currently affecting an estimated 7.1 million children under 18 years old, *in the U.S. according to W.H.O.*[14]. In the United States of America 9-11% children less than 18 years (9.5%) currently have asthma [15]. Asthma is a chronic condition involving the respiratory system in which the airways episodically constrict, become inflamed, and are lined with excessive amounts of mucus, often in response to one or more triggers [16].

These episodes may be triggered by environmental stimuli such as allergens, environmental tobacco smoke, cold or warm air, perfume, pet dander, moist air, exercise, or emotional stress. The resulting airway narrowing causes symptoms such as wheezing, shortness of breath, chest tightness, and coughing. Asthma is ideally treated by daily controller medications supplemented with short acting bronchodilators.

### The Biobehavioral aspects of asthma

**Figure 2:**
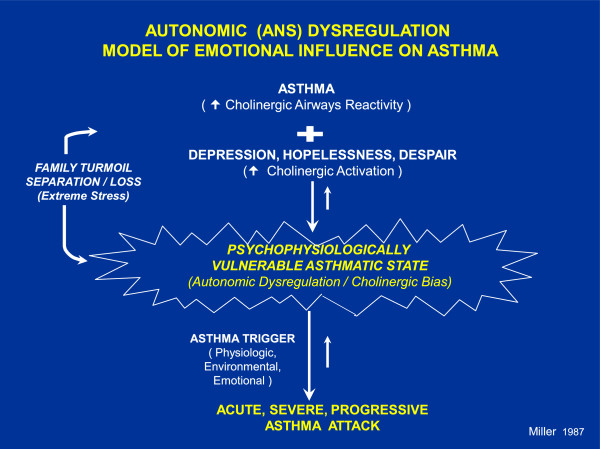
**Miller’s Autonomic Nervous System Dysregulation Model of the Effect of Emotions on Asthma.** (Wood and Miller, 2005).

*Asthma is caused by both environmental and genetic factors, the interaction of which is not fully understood*[17]*;* Onset of asthma in a child is likely determined by a complex interaction of genetic vulnerability, environmental exposure to respiratory infections, allergens, irritants, or environmental smoke and psychological influences such as maternal distress [18] and stress, and family dysfunction [1]. A theory positing direct family-psychobiologic pathways in asthma, such as the BBFM, must specify plausible psychobiologic mechanisms. Several have been empirically tested. The BBFM has been tested specifically with Miller’s Autonomic (ANS) Dysregulation Model. The model poses that chronic stress and depression accompanied by ANS dysregulation potentiates the effect of asthma triggers on airway function in asthma. (See Figure 2). *The model assumes that directions of causal effect are circular, but the current focus is on the child’s physical and emotional outcomes.*

## Case analysis and illustration

### Child and family assessment and formulation

The patient was at the time of treatment a 15-year-old girl. She lived in a rural area near Sparti in Greece with her biological parents and her two younger sisters (10 and 12 years old). Her parents were low income farmers, and financial problems stressed the family. The patient was brought to therapy by her parents, after recommendation of the family doctor, because her asthma was unstable, despite adhering to her asthma medication. The patient had severe asthma. She was treated with inhaled corticosteroids except for the time of crises, when she was treated with stronger medications, such as injections and bronchodilators, such as theophylline. Despite this aggressive treatment regimen, she still had frequent attacks, requiring emergency intervention and hospitalization. Her family doctor believed her to have life-threatening asthma.

The patient was isolated from friends and she felt quite ashamed of herself, because of her asthma attacks. She had poor grades, something that was a source of criticism from her parents. When she came to therapy she felt depressed, and thought that there was no solution to her problems.

The initial session was scheduled with the patient and her parents, who were unwilling to participate and preferred to wait in the waiting room until their daughter would be ‘examined’. They declared that they could not see any reason to visit a psychologist, and they came because the physician insisted they should come. According to the patient, they, like many Greek parents, thought that asthma was a totally physical disease with no psychological components. The parents refused to participate, so it was impossible for the therapist to treat the whole family. It is important to note that this family’s attitude and bias against psychologists and therapy was consistent with Greek cultural beliefs and attitudes in this realm. Such cultural attitudes are well documented in many research studies, including one that took place in 30 countries and was held by Georgas et al. [19].

Since the family was reluctant to participate in the sessions, and such reluctance was consistent with Greek attitudes, I believed that the success of therapy would be most supported by respecting these cultural beliefs and attitudes, rather than by challenging them. Furthermore, in my judgment, it was likely that the family would have taken their daughter out of treatment rather than submit themselves to therapy. Therefore, I had to rely upon the patient’s accounts and descriptions of her family.

We allow that there is always a subjective element introduced when a therapist tries to assess and /or understand a person’s or family’s attitude towards one another. However, even direct observation of family interaction occurs through a subjective lens. Consideration of such “limitations” is an epistemological debate. That is to say, some orientations would argue that it is the experience of the patient, as reported by the patient, that impacts on that person’s function, not what the “objective” observer would perceive and experience. Observation alone thus may not provide insight into how the interactions are experienced by the patient. Ideally both perspectives would be available. However in the absence of direct observation, one must listen carefully and broadly to the informant (the patient in this case) to establish convergent evidence for any perception, interpretation, assessment or conclusion about family function. This was the stance taken by the therapist in this case).

The treatment was guided by Wood’s BBFM and by Bowenian family intervention theory. Based on BBFM assessment, we observed the following:

#### Low proximity

The patient’s family did not communicate much. Her father was emotionally absent and quite critical, and perhaps depressed, as his income was quite low, and he had to accept his father-in-law’s financial help. The patient’s father’s depressive behavior contributed to her emotional difficulties. This observation was consistent with BBFM research findings, which demonstrated pathways of effects of paternal depression on the child.

The patient’s mother was compliant with almost every person in her family, but had sentiments of bitterness that she never expressed. It was forbidden in the family to express emotions or needs. The patient could not remember whether her parents had ever argued. The patient commented that they never shared emotions, even negative ones, since they rarely communicated openly, apart from the occasions when her father was critical towards her mother. It appeared from the patient’s descriptions that her parents had a very distant relationship, and were withdrawn and disengaged from interaction. They did not look at one another while talking, nor show facial expressiveness to one another. According to the patient, her father appeared not to listen when mother was talking to him. Throughout the sessions, the therapist learned of the patient’s impasse in trying to communicate with her parents, and her description of fruitless efforts to exchange opinions with her parents, who indeed, according to her description, did not speak other than to utter words necessary to accomplish the task at hand.

(For example, the therapist once asked if the patient could estimate the number of words uttered by her parents and/or exchanged between them and/or their daughter. Could they be more than a hundred per day? “Of course not, she replied, and the most of them were orders and or instructions followed by commands and/ or negative or ironic comments towards each other).

#### Weak generational hierarchy

At the same time, it appeared that an extreme “alliance” between the patient and her mother existed in which the patient became the “parentified”, “triangulated” child. The patient had observed that when the two parents were tired because of the verbal or non-verbal complaints they exchanged, they turned towards the patient and attributed to her responsibility for their fatigue. Alternatively, her mother would scapegoat the patient by complaining that her daughter accomplished her tasks very slowly. She wondered, “How the patient could ever be married one day with such a slow pace of housekeeping”.

In addition, there were no clear family boundaries or roles. Since her parents had a distant relationship with one another and others in the family, they lost control of the family. They could not provide care and nurturance to their children. The patient assumed a parental role. She was responsible for her two younger sisters when her parents worked in the fields. She had to nurture them, and prepare them for school. In addition to being parentified towards her siblings, she was also parentified towards her mother.

The patient shouldered the housework in order to protect her mother from her father’s criticism. Her mother made all the decisions for her family, but then received criticism when something did not work. The patient took steps to protect her mother from this, by making sure that everything in the house was working well, or by being extra supportive towards her mother. Her mother depended almost exclusively upon this young girl, who was unable to verbalize her negative feelings, mainly because of her fear that she might upset and emotionally distress her mother. There were also weak boundaries among the three generations. The patient’s father strongly aligned psychologically with his family of origin, which repeatedly “exploited” both him and his wife. Also, given that her mother’s father offered money to assist the household, he asked a great deal from the patient’s parents. In addition, her mother’s parents forced her parents implicitly –and sometimes explicitly- to vote and become active members of the political and social parties they belonged to. Furthermore, both grandparents imposed upon the patient’s parents to work for them on weekends, although they could have afforded someone else to help. Thus both parents became victims of “exploitation” (economically and psychologically) by their families of origin where neither limits nor personal boundaries existed. Roles were tragically divided between those of “exploiters” and “victims”. The patient’s parents were the “victims” of their parents’ “exploitation”, The patient’s mother was the “victim” of the father’s “exploitation”, and the patient was the “victim” of the mother’s “exploitation”.

#### Negative Inter-parental relationship

Since the spouses were alienated from each other, they avoided discussing matters that concerned them in order to maintain a barely tolerable relationship. The patient reported that she “felt” the negative emotional relationship between her parents. The lack of warmth, respect, and tenderness described seemed to contribute to the negative family emotional climate, which in turn added to the patient’s distress [8]. In addition, inter-parental emotional distance precluded mutual support, and undermined their being able to provide productive and caring parenting. These observations are consistent BBFM research findings.

#### Child neglect

Most of the time the patient was neglected with regard to her need for secure attachment and parental support. For example she was neglected in terms of her need to have a real discussion in a calm manner, to be supported in her first efforts to discuss her feelings towards her parents, and her intense feelings of loneliness and helplessness. Thus, the patient was functionally and essentially neglected, and insecurely attached to her parents, which stemmed from inadequate parenting. It was clear that no one cared, nor even suspected, that the stressful family situation overloaded the patient emotionally. These experiences over time contributed to a parent–child relational insecurity and distress in the child, which has been shown to contribute to asthma disease activity [1,8].

In contrast, when the patient became ill from asthmatic crises, her parents were really worried, and stopped neglecting her. They took good care of her, making sure that she would stay in bed and take her medication. These were the only occasions when the patient’s parents were united, and when other problems did not occupy their focus of attention. On the other hand, the patient’s asthmatic incidents caused further disruption of familial relationships. Her parents’ anxiety increased during asthmatic crises, because they were afraid lest their child would die. The patient could not breathe easily, and at times she could not breathe at all. Her parents were also tired because of lack of sleep, due to their staying awake next to their daughter’s bed. Also they were obliged to miss some days of work, because they could not pay for a nurse. These factors contributed to the family’s overall stress and instability, and subsequent neglect of the patient, which would, in turn, stress her and worsen her asthma in a cyclical spiral.

#### Inadequate family responsivity

The patient’s family thus fluctuated from extremely low levels of responsivity to extremely high levels of responsivity. The family members were psychologically and emotionally distant, only to be reunited and share their distress and worry after an asthma attack. There were also extreme levels of responsivity between the patient and her mother, which exacerbated maladaptive emotional and psychological resonance through emotional contagion [6]. The patient’s asthma attacks were more serious and more frequent when her mother’s negative emotions, depression and distress were elevated. This inference was based on time sequence of these events: if for example mother was in a depressive mood or rejected her directly or indirectly for days, or even hours, before her asthma attacks, it is reasonable to hypothesize that the stress of these relational events contributed to the asthma attack. There were frequent examples of these sequential events, all in the absence of other obvious triggers (e.g. allergens, infections, exercise). Taken together this became a pattern, which supports the inference of the effect of the mother’s depressed mood and negative interaction on the daughter’s asthma. This clinical observation was also consistent with research findings [6,8,11]. Taken together, the low proximity, weak generational hierarchy, and negative inter-parental relationship contributed to child neglect and to variable and maladaptive parent–child responsivity, all of which contributed to the patient’s emotional and physiological dysregulation (i.e. biobehavioral reactivity).

#### Biobehavioral reactivity

During difficult family times, the patient would become increasingly depressed, and she reported feeling “overwhelmed”. The patient’s emotional state was mainly depressive and hopeless. She felt lonely, misunderstood and unable to cope with specific situations. Research shows that these feelings are related to autonomic dysregulation accompanied by worse airway function and disease activity in child asthma [4,5]. After an attack, during which she had once again become a child who was nurtured and cared for, the patient’s emotions would stabilize and improve, and asthma attacks would disappear for long periods of time.

The patterns of BBFM relational stress described above, provided the formulation of the patient’s family contextual situation, and guided the intervention process with clear direction and focus.

#### Intervention

The patient was treated by a physician for asthma and by a psychologist (first author) for her emotional and family difficulties. The psychotherapy lasted 6 months, with weekly sessions, followed by planned follow-ups. *The long-term asthma control medications included inhaled corticosteroids (Pulmicort), leukotriene modifiers (singulair),combination inhalers (Symbicort) and Theophylline daily. These medications were judged as the most effective medications in order to keep asthma under control. Before the psychotherapeutic intervention, she had often acute severe asthmatic crises, during which she ought to go either to clinic or in the hospital. She ought to be hospitalized, be injected and receive oral corticosteroids. After counseling, she learned to set limits and manage herself, so that she could prevent asthmatic crises. Thus, she was neither hospitalized nor she receive oral corticosteroids during and after psychological counseling.*

Since the family was unwilling to participate, the therapist used Bowenian theories [3] and intervention strategies, which lend themselves well to individual treatment while addressing family relational aspects of the patient’s difficulties. The therapist sought to help the adolescent see things differently and develop competence and confidence, and guided her in disentangling herself from the family’s maladaptive patterns while developing her own fulfilling adolescent life. She also intervened to increase the patient’s differentiation. This served to reduce her vulnerability to the problematic family processes.

The treatment addressed specific dimensions of BBFM that were particularly problematic for the patient. For example, her parents’ troubled relationship was discussed, and the patient was helped to realize that it was not appropriate for her to buffer her mother from her father. D. was helped with this by elaborating on concrete examples derived from her own descriptions: were her interventions effective? No… How did her interventions influence her parents’ interactions? They did not… This line of inquiry helped the patient to realize that her interventions were not effective. She was also helped to realize that it is not appropriate by coming to realize that it was damaging to herself, and that it is not a teenager’s responsibility to sacrifice her health for her parents, especially if it was not helping them. Thus, the triangulation was challenged, and it began to diminish, and a process of self-differentiation was shaped, encouraged and reinforced (addressing the effect of inter-parental negativity and triangulation of the child).

To support the patient’s differentiation and to decrease the effect of the depressing family emotional climate, the therapist focused on appropriate adolescent developmental tasks. Adolescence is a key time in a young woman’s life. She needs to be encouraged to form her own identity and life. In Greece, it is very difficult, and not expected, for a young adult also to be completely independent financially, which may undermine adolescent *differentiation*. Nonetheless, Greek families do typically help their adolescents and young adults achieve differentiated functioning [19]. However, when a family, such as the patient’s, shows vague interpersonal boundaries; no strong sense of individuality is possible. This condition is reminiscent of what Bowen has described as an undifferentiated family ego mass.

It is important to note that there are additional cultural aspects to take into account in assisting an adolescent to “differentiate”, For example, in some cultures family members may “violate” each other’s boundaries without problematic outcomes. For example, boundary invasion is usually observed in Greek families. In Greece *“individuality” has not been considered a virtue for hundreds of years, unlike in the US where is held in high value*[19]. In conclusion culturally relative differentiation is the appropriate aim in a therapeutic encounter, rather than diffentiation in absolute terms.

The patient was helped to introduce amusement activities for herself into her weekly program. She started to go to the pool and to take care of herself while avoiding being caught in triangulations. Her family unfortunately, but predictably, resisted the patient’s differentiation. They made comments such as: “Nowadays, children do not respect their parents. You have started recently to become rude and selfish, as if you did not know how many problems we face. If you do not care for your family, who will?”

This resistance influenced the young girl negatively, as she was inspired to help her parents and sisters to live a more fulfilling life despite their problems. The therapist supported the patient ***in setting boundaries***, in concert with an attitude of respect towards her parents, in the face of these challenges.

At the same time, occasions in which the crises occurred were thoroughly studied by the patient and the therapist in collaboration. They addressed “process questions”, with regard to the familial relations and the role of patient in the mesh of these relations. Questions raised were: “If your mother had a dispute with your father or sister, how would you deal with it?” “Did she ask you to comfort her or did she blame you for not having helped before?” “How did you feel inside?” “What did you do?” Or “When your father blamed his parents-in–law for causing a conflict, what went on inside you?” The patient was assisted in observing that a correlation existed between the depression and the despair that she was experiencing, and the occurrence of asthma crises. The patient also gradually came to realize that she was neither responsible for, nor did she have the capacity to resolve, the unsolvable problems of her family. She also came to accept that it was not her duty to provide solutions to, or exclusively support, her mother (addressing in appropriate emotional responsivity, biobehavioral reactivity, and weak generational hierarchy).

Another objective was to psychologically support and to reinforce the patient’s *differentiation*. She was assisted in expressing her distress and despair verbally, at least during sessions. Realizing and verbalizing her feelings helped her to start observing herself and her interactions with her family. Also, it was important for her to be able to determine her “self space” and her personal limits and to decrease her guilt and self-blame. Through trial and error assignments, she was encouraged to question her own maladaptive patterns. For example, “was the feeling of constant guilt for her behavior towards her unsatisfied parents a useful attitude?” “Could she become less afraid of her parents’ reactions?” She was assigned small experiments in order to test, herself, the consequences of her relatedness to her internal and external emotional environment (addressing inappropriate responsivity, strengthening emotion regulation, and decreasing biobehavioral reactivity).

The concept of Biobehavioral reactivity helped the patient a great deal. The concept enabled her to understand and identify her “psychosomatic” limits. She started writing a diary, where she noted the internal and external events that preceded and succeeded the asthmatic crises. She was encouraged to take a distance and study the interactions’ sequencing. A very effective technique here to encourage differentiation was the technique of writing a diary. This technique was suggested by my supervisor (Bowenian therapist Dr. N. Charitos), and is amply evidenced in Dr. Penn’s approach [20] to family therapy.

This helps the person to realize a personal internal space’s existence, space that usually is absent in ‘enmeshed’ families, as Minuchin describes them [2] It also reduces the inappropriate “responsivity” as defined in the BBFM [4].

These self-observations fostered a clear view of her sensitivity, her personal limits with regard to her asthmatic crises. These changes were neither easy nor automatic. The patient was caught in a pattern of being the problem- solver for her family. It was difficult for her to relinquish this role, but changes did occur in small steps (addressing weak generational hierarchy).

Simultaneously, re-thinking her life style, and examining her feelings from a more rational perspective, contributed to her increasing competence, self- awareness and *differentiation of herself.* Thus, she became able to assertively explain why she chose to say “no” to her family in certain circumstances. She expressed respect towards her parents and caring towards her sisters, but she explained that it was beyond her physical abilities to support her family at the expense of her health. Writing down her observations regarding the relationships in her family helped her realize the triangles she was involved in with her parents, and how the weak boundaries between her parents and her grandparents created a ‘togetherness amalgam’, in which she was entangled. It took quite a bit of time before the patient could set limits, and dare speak up for herself to her family, when they intrusively sought inappropriate support from her. Successful treatment requires substantial experiential practice, because insight is not sufficient to evoke lasting change [21,22].

The follow-ups were every two weeks for the first year and every month for the second year. Sessions focused on helping the young adolescent stabilize her new differentiation and her improved psychosocial adjustment. At the end of treatment, the patient’s asthmatic symptoms were controlled, so that she could avoid asthmatic crises and hospitalization. Moreover she was emotionally content and engaging in developmentally appropriate activities. Her family was still a source of sadness and stress for her, but she was able to sustain her own physical and emotional well being without severing her ties with her family.

## Conclusion

In this case study, the family patterns observed and the manner in which they impacted on the patient’s emotional and physical compromise were consistent with the findings of research testing the BBFM. The fact that the patient’s health improved by means of psychotherapy, focusing on these family patterns without any changes in her medical-pharmaceutical care, constituted another piece of evidence for the important role of these factors in the development and course of illness. *Of course, in the above mentioned conclusions there are limitations, which are due to the fact that all the observations are based on a single case study.*

*Given these limitations, we think that* that a biopsychosocial approach is necessary in the treatment of diseases *in which there is a psychobiological pathway*. Such approaches could “break” the vicious cycle of maladaptive biopsychosocial interactions, thus yielding better therapeutic results, without a need for an increase in the dosage and side effects of medication. The BBFM provides specific targets upon which to focus a biopsychosocial intervention.

Note: Term definition:

“Stress” is the organism’s response to stressful conditions or stressors, consisting of a pattern of physiological and psychological reactions, both immediate and delayed.

“Onset of illness” is defined by the appearance of clinical symptoms of disease.

“Predisposing factors” are longstanding behavior patterns, childhood experiences, and durable personal and social characteristics that may alter the susceptibility of the individual to illness.

“Precipitating factors,” in contrast, influence the timing of illness onset; the term refers for the most part to more or less transient changes in current conditions or characteristics, and it is such changes that constitute our present subject of inquiry.

“Chronic disease” refers here very generally to syndromes which are of long duration and are noninfectious.

## Consent

“Written informed consent was obtained from the patient for publication of this Case report and any accompanying images. A copy of the written consent is available for review by the Editor-in-Chief of this journal”.

## Competing interests

The authors declare that they have no competing interests.

## Author’s contributions

MT conducted the treatment, conceptualized the presentation of the model as case study and wrote the main parts of the article. OA and PA discussed treatment and the application of the model to the case with the first author. Prof. BW participated with the first author in the written conceptualization of the presentation of the model as applied to the case. All authors read and approved the final manuscript.“ Dr. BW participation on the study was partially supported by a grant (1R01MH064154) from National Institute of Health, USA.
